# 2-Amino-5-methyl­pyridinium dibromo­iodate

**DOI:** 10.1107/S1600536812036136

**Published:** 2012-08-25

**Authors:** Salim F. Haddad, Basem F. Ali, Rawhi Al-Far

**Affiliations:** a Department of Chemistry, The University of Jordan, Amman 11942, Jordan; b Department of Chemistry, Al al-Bayt University, Mafraq 25113, Jordan; c Faculty of Science and IT, Al-Balqa’a Applied University, Salt, Jordan

## Abstract

In the title salt, C_6_H_9_N_2_^+^·Br_2_I^−^, the cation is essentially planar (r.m.s. deviation = 0.0062 Å for the non-H atoms) while the anion is almost linear with a Br—I—Br angle of 177.67 (2)°. The crystal packing shows two anions and two cations connected *via* N—H⋯Br and (pyridine)N—H⋯Br hydrogen-bonding inter­actions, forming centrosymmetric tetra­mers with *R_4_^4^*(16) ring motifs. Very weak offset aromatic π–π stacking interactions [centroid-centroid separation = 4.038 (4), slippage = 1.773 Å] also occur.

## Related literature

For background to this study, see: Al-Far *et al.* (2012 ▶); Kochel (2006 ▶). For comparison bond lengths and angles, see: Gardberg *et al.* (2002 ▶); Hemamalini & Fun (2010 ▶). For graph-set notation, see: Bernstein *et al.* (1995 ▶). 
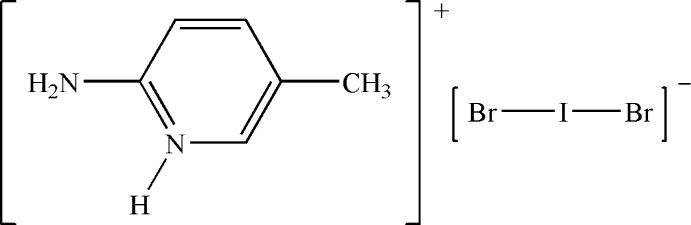


## Experimental

### 

#### Crystal data

C_6_H_9_N_2_^+^·Br_2_I^−^
*M* *_r_* = 395.85
Triclinic, 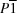

*a* = 8.3648 (13) Å
*b* = 8.4233 (16) Å
*c* = 9.2321 (16) Å
α = 105.107 (16)°
β = 115.371 (16)°
γ = 98.241 (15)°
*V* = 542.7 (2) Å^3^
*Z* = 2
Mo *K*α radiation
μ = 10.26 mm^−1^
*T* = 293 K
0.54 × 0.39 × 0.30 mm

#### Data collection

Agilent Xcalibur Eos diffractometer
Absorption correction: multi-scan (*CrysAlis PRO*; Agilent, 2011 ▶) *T*_min_ = 0.011, *T*_max_ = 0.045
4283 measured reflections
2465 independent reflections
1777 reflections with *I* > 2σ(*I*)
*R*_int_ = 0.029

#### Refinement

*R*[*F* ^2^ > 2σ(*F* ^2^)] = 0.040
*wR*(*F* ^2^) = 0.096
*S* = 1.01
2465 reflections
102 parameters
H-atom parameters constrained
Δρ_max_ = 1.17 e Å^−3^
Δρ_min_ = −0.85 e Å^−3^

### 

Data collection: *CrysAlis PRO* (Agilent, 2011 ▶); cell refinement: *CrysAlis PRO*; data reduction: *CrysAlis PRO*; program(s) used to solve structure: *SHELXS97* (Sheldrick, 2008 ▶); program(s) used to refine structure: *SHELXL97* (Sheldrick, 2008 ▶); molecular graphics: *SHELXTL* (Sheldrick, 2008 ▶); software used to prepare material for publication: *SHELXTL*.

## Supplementary Material

Crystal structure: contains datablock(s) I, global. DOI: 10.1107/S1600536812036136/pv2581sup1.cif

Structure factors: contains datablock(s) I. DOI: 10.1107/S1600536812036136/pv2581Isup2.hkl

Additional supplementary materials:  crystallographic information; 3D view; checkCIF report

## Figures and Tables

**Table 1:** Hydrogen-bond geometry (Å, °)

*D*—H⋯*A*	*D*—H	H⋯*A*	*D*⋯*A*	*D*—H⋯*A*
N1—H1*A*⋯Br2	0.86	2.73	3.499 (5)	150
N2—H2*B*⋯Br1^i^	0.86	2.70	3.545 (6)	168

Symmetry code: (i) 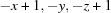.

**Fig. 1.**
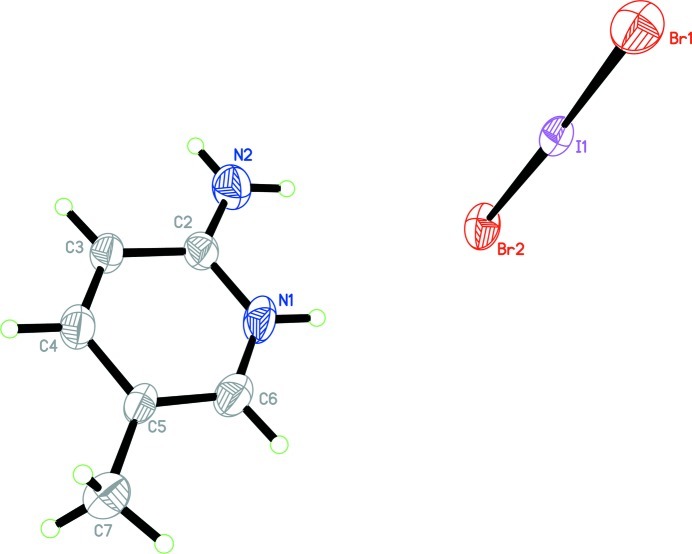
The molecular structure of the title compound with the atom numbering scheme. Displacement ellipsoids are drawn at the 30% probability level. H atoms are presented as small spheres of arbitrary radius.

**Fig. 2.**
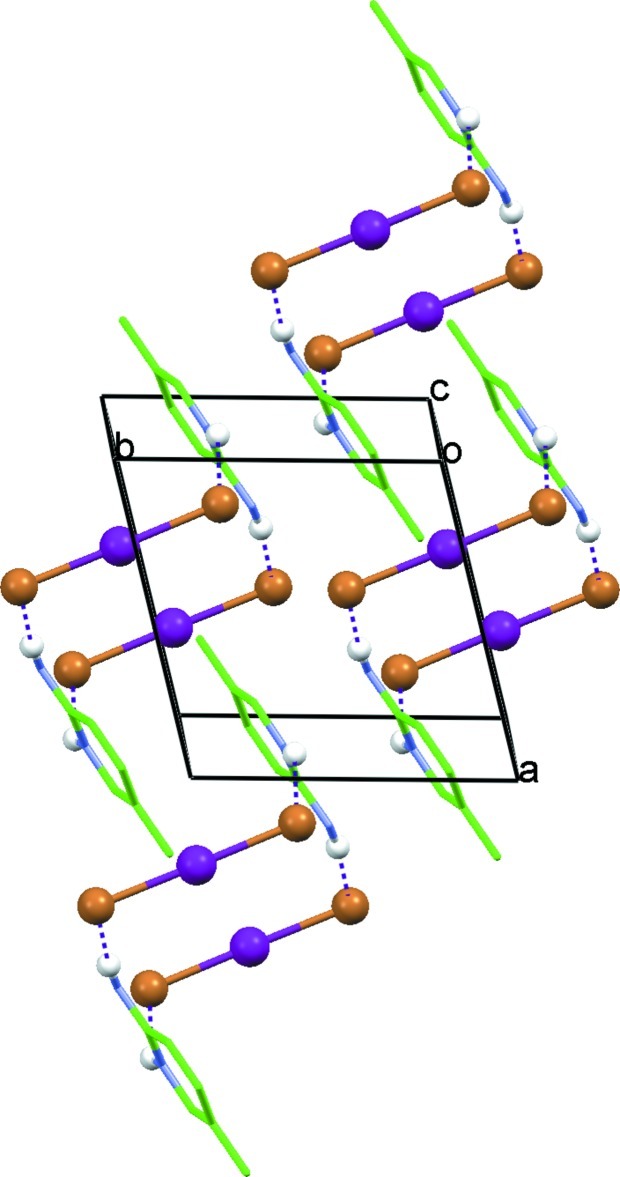
A view of the pyN–H···Br and H–N–H···Br hydrogen bonds (dotted lines) in the crystal structure of the title compound. H atoms non-participating in hydrogen-bonding were omitted for clarity.

## supplementary crystallographic information

### Comment

Polyhalides display a variety of structures. Various compounds with interesting structures were found when protonated aromatic nitrogen bases were combined with polyhalides (Kochel, 2006). Continuing our research in this area (Al-Far *et al.*, 2012), we now report the crystal structure of the title compound in this article. The cystals of the title compound were found as an unexpected product from a reaction mixture of CdI_2_, HBr, 2-amino-5-methylpyridine and Br_2_ upon attempting to synthesize [(C_7_H_10_N)]_2_ [CdBr_4_] complex of 2-amino-5-methylpyrinium.

In the title compound (Fig. 1), the cation, 2-amino-5-methylpyridinium, is essentially planar (r.m.s.d = 0.0062 Å). The IBr_2_^-^ anion is symmetrical and almost linear, Br1—I—Br2 angle of 177.67 (2) °, with I—Br distances 2.6836 (10) and 2.7119 (10) Å. These values are in agreement with the values reported in the literature (Gardberg *et al.*, 2002). The molecular dimensions of the cation are also as expected (Hemamalini & Fun, 2010).

The crystal structure (Fig. 2), shows stacks of anions separated by layers of cations. The anions and cations are connected *via* H–N–H···Br and pyN–H···Br hydrogen bonding (Table 1), forming centrosymmetric tetramers (two cation and two anions). These tetramers form sixteen membered rings in graph set motif *R*_4_^4^(16) (Bernstein *et al.*, 1995). The rings are further connected *via*π···π interactions between the cations with separation betweeen the ring centroids [*C_g_*···*C_g_* (2 - *x*, -*y*, 1 - *z*)] being 4.038 (4) Å. Both hydrogen bonding and π···π interactions consolidate a three dimensional network.

### Experimental

A solution of CdI_2_ (0.37 g, 1.0 mmol) dissolved in 95% EtOH (10 ml) and 60% HBr (1 ml) solution was added to a mixture of 2-amino-5-methylpyridine (0.11 g, 1.0 mmol) dissolved in 95% EtOH (10 ml), 60% HBr (1 ml) and molecular bromine (2 ml). The resulting mixture was refluxed for 2.5 hr. On slow evaporation at room temperature yellow plates of the title compound were formed in 4 days (yield 85%).

### Refinement

All H atoms were positioned geometrically and refined using a riding model, with N—H = 0.86 Å and C—H = 0.93 and 0.96 Å, for aryl and methyl H-atoms, respectively. The *U*_iso_(H) were allowed at 1.5*U*_eq_(C methyl) or 1.2*U*_eq_(N/C non-methyl).

### Crystal data

**Table:**

C_6_H_9_N_2_^+^·Br_2_I^−^	*Z* = 2
*M**_r_* = 395.85	*F*(000) = 364
Triclinic, *P*1	*D*_x_ = 2.422 Mg m^−^^3^
Hall symbol: -P 1	Mo *K*α radiation, λ = 0.71073 Å
*a* = 8.3648 (13) Å	Cell parameters from 1406 reflections
*b* = 8.4233 (16) Å	θ = 3.2–30.0°
*c* = 9.2321 (16) Å	µ = 10.26 mm^−^^1^
α = 105.107 (16)°	*T* = 293 K
β = 115.371 (16)°	Plate, yellow
γ = 98.241 (15)°	0.54 × 0.39 × 0.30 mm
*V* = 542.7 (2) Å^3^	

### Data collection

**Table:**

Agilent Xcalibur Eos diffractometer	2465 independent reflections
Radiation source: Enhance (Mo) X-ray Source	1777 reflections with *I* > 2σ(*I*)
Graphite monochromator	*R*_int_ = 0.029
Detector resolution: 16.0534 pixels mm^-1^	θ_max_ = 29.1°, θ_min_ = 3.2°
ω scans	*h* = −11→10
Absorption correction: multi-scan (*CrysAlis PRO*; Agilent, 2011)	*k* = −11→11
*T*_min_ = 0.011, *T*_max_ = 0.045	*l* = −10→12
4283 measured reflections	

### Refinement

**Table:**

Refinement on *F*^2^	Secondary atom site location: difference Fourier map
Least-squares matrix: full	Hydrogen site location: inferred from neighbouring sites
*R*[*F*^2^ > 2σ(*F*^2^)] = 0.040	H-atom parameters constrained
*wR*(*F*^2^) = 0.096	*w* = 1/[σ^2^(*F*_o_^2^) + (0.035*P*)^2^] where *P* = (*F*_o_^2^ + 2*F*_c_^2^)/3
*S* = 1.01	(Δ/σ)_max_ < 0.001
2465 reflections	Δρ_max_ = 1.17 e Å^−^^3^
102 parameters	Δρ_min_ = −0.85 e Å^−^^3^
0 restraints	Extinction correction: *SHELXL97* (Sheldrick, 2008), Fc^*^=kFc[1+0.001xFc^2^λ^3^/sin(2θ)]^-1/4^
Primary atom site location: structure-invariant direct methods	Extinction coefficient: 0.0292 (12)

### Special details

**Table:**

Geometry. All e.s.d.'s (except the e.s.d. in the dihedral angle between two l.s. planes) are estimated using the full covariance matrix. The cell e.s.d.'s are taken into account individually in the estimation of e.s.d.'s in distances, angles and torsion angles; correlations between e.s.d.'s in cell parameters are only used when they are defined by crystal symmetry. An approximate (isotropic) treatment of cell e.s.d.'s is used for estimating e.s.d.'s involving l.s. planes.
Refinement. Refinement of *F*^2^ against ALL reflections. The weighted *R*-factor *wR* and goodness of fit *S* are based on *F*^2^, conventional *R*-factors *R* are based on *F*, with *F* set to zero for negative *F*^2^. The threshold expression of *F*^2^ > σ(*F*^2^) is used only for calculating *R*-factors(gt) *etc*. and is not relevant to the choice of reflections for refinement. *R*-factors based on *F*^2^ are statistically about twice as large as those based on *F*, and *R*- factors based on ALL data will be even larger.

### Fractional atomic coordinates and isotropic or equivalent isotropic displacement parameters (Å^2^)

**Table:**

	*x*	*y*	*z*	*U*_iso_*/*U*_eq_	
N1	1.0416 (7)	0.3049 (7)	0.7106 (6)	0.0606 (15)	
H1A	1.0387	0.3331	0.8057	0.073*	
I1	0.70649 (5)	−0.04784 (5)	0.90281 (5)	0.04214 (17)	
Br1	0.55742 (10)	−0.38624 (9)	0.80942 (10)	0.0661 (2)	
N2	0.7745 (7)	0.3836 (7)	0.5894 (7)	0.0699 (17)	
H2A	0.7751	0.4121	0.6862	0.084*	
H2B	0.6882	0.3947	0.5021	0.084*	
C2	0.9069 (8)	0.3223 (8)	0.5748 (8)	0.0509 (16)	
Br2	0.85761 (10)	0.29148 (9)	0.98508 (9)	0.0589 (2)	
C3	0.9133 (8)	0.2686 (8)	0.4207 (8)	0.0500 (15)	
H3A	0.8224	0.2749	0.3211	0.060*	
C4	1.0533 (8)	0.2076 (9)	0.4194 (8)	0.0551 (17)	
H4A	1.0562	0.1728	0.3166	0.066*	
C5	1.1936 (8)	0.1936 (8)	0.5625 (7)	0.0438 (14)	
C6	1.1819 (9)	0.2455 (9)	0.7061 (9)	0.0591 (18)	
H6A	1.2732	0.2406	0.8064	0.071*	
C7	1.3488 (8)	0.1264 (9)	0.5598 (9)	0.0647 (19)	
H7A	1.4329	0.1335	0.6734	0.097*	
H7B	1.4130	0.1938	0.5211	0.097*	
H7C	1.2999	0.0087	0.4830	0.097*	

### Atomic displacement parameters (Å^2^)

**Table:**

	*U*^11^	*U*^22^	*U*^33^	*U*^12^	*U*^13^	*U*^23^
N1	0.077 (4)	0.061 (4)	0.032 (3)	0.007 (3)	0.025 (3)	0.008 (3)
I1	0.0468 (3)	0.0479 (3)	0.0301 (2)	0.01598 (19)	0.01656 (19)	0.01427 (19)
Br1	0.0739 (5)	0.0464 (4)	0.0580 (5)	0.0088 (4)	0.0210 (4)	0.0132 (4)
N2	0.076 (4)	0.076 (5)	0.060 (4)	0.019 (3)	0.039 (3)	0.019 (4)
C2	0.052 (3)	0.050 (4)	0.046 (4)	0.002 (3)	0.023 (3)	0.018 (3)
Br2	0.0794 (5)	0.0461 (4)	0.0465 (4)	0.0117 (4)	0.0284 (4)	0.0173 (4)
C3	0.054 (4)	0.054 (4)	0.038 (4)	0.011 (3)	0.023 (3)	0.014 (3)
C4	0.062 (4)	0.058 (4)	0.041 (4)	0.008 (3)	0.026 (3)	0.014 (3)
C5	0.049 (3)	0.042 (4)	0.031 (3)	0.005 (3)	0.013 (3)	0.015 (3)
C6	0.060 (4)	0.064 (5)	0.040 (4)	0.013 (4)	0.016 (3)	0.016 (4)
C7	0.063 (4)	0.069 (5)	0.057 (5)	0.022 (4)	0.023 (4)	0.025 (4)

### Geometric parameters (Å, º)

**Table:**

N1—C2	1.340 (7)	C3—H3A	0.9300
N1—C6	1.352 (8)	C4—C5	1.389 (8)
N1—H1A	0.8600	C4—H4A	0.9300
I1—Br1	2.6836 (10)	C5—C6	1.334 (8)
I1—Br2	2.7119 (10)	C5—C7	1.496 (8)
N2—C2	1.330 (7)	C6—H6A	0.9300
N2—H2A	0.8600	C7—H7A	0.9600
N2—H2B	0.8600	C7—H7B	0.9600
C2—C3	1.402 (8)	C7—H7C	0.9600
C3—C4	1.348 (8)		
			
C2—N1—C6	123.5 (5)	C3—C4—H4A	118.0
C2—N1—H1A	118.3	C5—C4—H4A	118.0
C6—N1—H1A	118.3	C6—C5—C4	115.2 (6)
Br1—I1—Br2	177.67 (2)	C6—C5—C7	121.3 (6)
C2—N2—H2A	120.0	C4—C5—C7	123.5 (5)
C2—N2—H2B	120.0	C5—C6—N1	122.1 (6)
H2A—N2—H2B	120.0	C5—C6—H6A	118.9
N2—C2—N1	120.1 (6)	N1—C6—H6A	118.9
N2—C2—C3	123.6 (6)	C5—C7—H7A	109.5
N1—C2—C3	116.3 (6)	C5—C7—H7B	109.5
C4—C3—C2	118.9 (6)	H7A—C7—H7B	109.5
C4—C3—H3A	120.5	C5—C7—H7C	109.5
C2—C3—H3A	120.5	H7A—C7—H7C	109.5
C3—C4—C5	123.9 (6)	H7B—C7—H7C	109.5
			
C6—N1—C2—N2	−179.2 (6)	C3—C4—C5—C6	0.0 (10)
C6—N1—C2—C3	2.4 (9)	C3—C4—C5—C7	−179.7 (6)
N2—C2—C3—C4	−179.7 (6)	C4—C5—C6—N1	1.0 (10)
N1—C2—C3—C4	−1.4 (9)	C7—C5—C6—N1	−179.3 (6)
C2—C3—C4—C5	0.3 (10)	C2—N1—C6—C5	−2.3 (10)

### Hydrogen-bond geometry (Å, º)

**Table:**

*D*—H···*A*	*D*—H	H···*A*	*D*···*A*	*D*—H···*A*
N1—H1*A*···Br2	0.86	2.73	3.499 (5)	150
N2—H2*B*···Br1^i^	0.86	2.70	3.545 (6)	168

Symmetry code: (i) −*x*+1, −*y*, −*z*+1.
